# Real-Time Detection and Measurement of Eye Features from Color Images

**DOI:** 10.3390/s16071105

**Published:** 2016-07-16

**Authors:** Diana Borza, Adrian Sergiu Darabant, Radu Danescu

**Affiliations:** 1 Computer Science Department, Technical University of Cluj Napoca, 28 Memorandumului Street, Cluj Napoca 400114, Romania; borza_diana@yahoo.com; 2 Computer Science Department, Babes Bolyai University, 58-60 Teodor Mihali, C333, Cluj Napoca 400591, Romania; adrian.darabant@tvarita.ro

**Keywords:** iris segmentation, sclera segmentation, eye center localization, shape analysis, eyelid segmentation

## Abstract

The accurate extraction and measurement of eye features is crucial to a variety of domains, including human-computer interaction, biometry, and medical research. This paper presents a fast and accurate method for extracting multiple features around the eyes: the center of the pupil, the iris radius, and the external shape of the eye. These features are extracted using a multistage algorithm. On the first stage the pupil center is localized using a fast circular symmetry detector and the iris radius is computed using radial gradient projections, and on the second stage the external shape of the eye (of the eyelids) is determined through a Monte Carlo sampling framework based on both color and shape information. Extensive experiments performed on a different dataset demonstrate the effectiveness of our approach. In addition, this work provides eye annotation data for a publicly-available database.

## 1. Introduction

The eyes and their movements can be used as an unobtrusive method to gain deeper understanding of one’s cognitive and neurological processes: studies show that facial expressions are the major interaction modality in human communication, as they contribute for 55% to the meaning of the message. Robust eye detection and tracking algorithms are crucial for a wide variety of disciplines, including human computer interaction, medical research, optometry, biometry, marketing research, and automotive design (notably through driving attention monitoring systems).

In today’s context of globalization, security is becoming more and more important. Biometry refers to a set of methods that rely on a set of distinctive, measurable characteristics that are used to describe individuals for the purpose of identification and access control. As opposed to traditional methods of identification (token-based identification or knowledge-based identification), biometric data is unique and permanently associated with an individual.

Iris recognition is growing in popularity and is currently being used in a broad set of applications. The first step in any iris recognition process is the accurate segmentation of the region of interest: the localization of the inner and outer boundaries of the iris and removal of eyelids, eyelashes, and any specular reflections that may occlude the region of interest. Secondly, the iris region is transformed into a rubber-sheet model and a unique bit pattern encoding is computed based on these pixels. 

Recently, a new ocular biometric technique has received particular interest from the scientific community: sclera recognition [1]. The sclera region (the white outer coat of the eye) comprises a unique and stable blood vessel structure which can be analyzed to identify humans. Moreover, this method can complement iris biometry by increasing its performance in case of off-angle or off-axis images. 

The major challenges that ocular biometry poses is the accurate segmentation of the regions of interest (the iris or the sclera). An incorrect segmentation can reduce the available information, but it can also introduce other patterns (eyelashes, eyelids) that can influence the biometric applicability of the trait.

This work focuses on the accurate segmentation of the eye: the iris region and the external shape of the eye (the eyelids). Therefore, this paper’s results are relevant in a variety of domains, ranging from iris and sclera biometrics, psychology, psychiatry, optometry, education, and driving assistance monitoring.

This paper will highlight the following contributions:

- A fast and accurate iris localization and segmentation algorithm;
- A new method for representing the external shape of the eyes based on six control points used to generate two parabolas for the upper and lower eyelids;
- An original sclera segmentation algorithm based on both color and shape constraints (as opposed to other methods from the specialized literature which are based solely on color information [2,3,4]);
- A Monte Carlo segmentation algorithm based on the proposed model and the corresponding matching methodology; and
- The annotation [5] of a publicly-available database with the eye positions and eyelid boundaries.

The rest of this paper is organized as follows. Section 2 presents a general overview of the most relevant research literature on eye localization, iris and sclera segmentation. The proposed method is detailed in Section 3. Experimental results on eye localization and segmentation are presented in Section 4. The conclusions and future research directions are presented in Section 5.

## 2. Related Work

Over the past decades eye localization and tracking has been one of the most active research areas in the field of computer vision, mainly due to its application to a wide variety of research fields. The organization of all the eye localization methods into a single and general taxonomy proves to be a challenging task.

Based on the light source, eye detection methods can be classified as *active light* methods (that rely on infrared or near-infrared light sources) and *passive light* methods (that use visible light to illuminate the eyes). Two general types of active light method can be exploited using a physical property of the pupil that modifies its appearance in the captured image depending on the position of the IR illuminator: *bright pupil* and *dark pupil*. When the IR illuminator is coaxial with the camera, the pupil appears as a bright zone (bright pupil), and when the light source is offset from the optical path the pupil appears dark (dark pupil). This work uses a passive light method.

In [6] a detailed survey on eye detection and tracking techniques is presented, with emphasis on the challenges they pose, as well as on their importance in a broad range of applications. The authors propose a three class taxonomy based on the model used to represent the eyes: *shape-based* methods, *appearance-based* methods, and *hybrid* methods.

Shape-based methods use a prior model of the eye geometry and its surrounding texture and a similarity measure. Appearance-based methods detect and track eyes based on the color distribution or filter responses of the eye region. These methods require a large amount of training data representing the eyes under different illumination conditions and face poses. 

Finally, hybrid methods combine two or more approaches to exploit their benefits and to overcome their drawbacks. As an example, the constrained local models (CLM) [7] framework, a recently emerged promising method for facial feature detection, uses a shape model to constrain the location where each feature might appear and a patch model to describe the appearance of the feature. In [8] a hybrid method is proposed to extract features of the human face. First, a Viola-Jones face detector is applied to approximate the location of the face in the input image. Next, individual facial features detectors are evaluated and combined based on shape constraints. Finally, the results are refined using active appearance models tuned for edge and corner cues. A three-stage facial feature detection method is presented in [9]. On the first stage, the face region is localized based on the Hausdorff distance between the edges from the input image and a template of facial edges. The second stage uses a similar, smaller model for the eyes. Finally, the pupil locations are refined using a multi-layer perceptron trained with pupil centered images.

Other methods localize several features on the face using local landmark descriptors and a predefined face shape model. In [10] the authors propose a new approach to localize features in facial images. Their method uses local detectors for each feature and combines their output with a set of global models for the part locations computed from a labeled set of examples. To model the appearance of each feature, a sliding window detector based on support vector machine (SVM) classifier with gray-scale scale invariant feature transform (SIFT) features is used. The work presented in [11] localizes nine facial landmarks in order to investigate the problem of automatic labeling of characters in movies. In the first stage, the position and the approximate scale of the face is detected in the input image. The appearance of each feature is determined using a variant of the AdaBoost classifier and Haar-like image features. Next, a generative model of the feature positions is combined with the appearance score to determine the exact position of each landmark.

The approach presented in our paper uses a fast shape based approach to locate the center of the irises. After the accurate iris segmentation, the external shape of the eyes (the eyelids) is segmented based on color information and shape constraints. The entire method takes, on average, 20 ms per processed image.

In general shape-based eye localization techniques impose a circularity shape constraint to detect the iris and the pupils [12,13], making the algorithms suitable only for near-frontal images.

In [12], the authors developed a fast eye center localization based on image gradients: the center of the circular pattern of the iris is defined as the region where most of the image gradients intersect. An additional post-processing step, based on prior knowledge of the appearance of the eyes, is applied to increase the robustness of the method on images where the contrast between the sclera and the iris is not that obvious, and occlusions (hair, eyelashes, glasses, etc.) are present.

In [14] a multi-stage circular Hough transformation is used to determine the iris center and its radius. Additionally, a similarity measure for selecting the iris center is computed based on the circularity measure of the Hough transform, the distance between the hypothetical iris centers and the contrast between the presumed iris and its background.

In [15] the eyes are localized using two distinctive features of the iris compared to the surrounding area: the eye region has an unpredictable local intensity and the iris is darker compared to the neighboring pixels. The eye centers are selected using a score function based on the entropy of the eye region and the darkness of the iris.

With the development of ocular biometrics, accurate localization of the iris and pupil area has drawn the attention of computer vision scientists. Iris and pupil segmentation were pioneered by Daugman [13] who proposed a methodology that is still actual: an integro-differential operator which uses a circular integral to search for the circular path where the integral derivative is maximal. The method searches for the circular contour that generates the maximum intensity change in pixel values by varying the center (*c_x_, c_y_*) and the radius (*radii*) of the path. This operator has high computational complexity (for each possible center multiple radii scans are necessary to compute this operator) and it has problems detecting the iris boundary in cases of low intensity separability between the iris and the sclera region. Another influential approach [16] uses a two-stage iris segmentation method: first, a gradient based binary edge map is created, followed by a circular Hough transform to find the parameters of the iris circle. The binary map is generated so that it favors ranges of orientation (for example, to delimit the iris-sclera region, image derivatives are weighted to be more sensible to vertical edges). The main disadvantage of this method is its dependence on the threshold values used in the edge map construction phase.

Other shape based eye detection studies use a more complex model of the eye [17,18] by also modeling the eyelids, but they are computationally demanding and their performance is strongly linked to the initial position of the template. In [18] the eyes are extracted using a deformable template which consists of a circle for describing the iris and two parabolas for the upper and lower eyelid. The template is matched over the input image using energy minimization techniques. A similar approach is used in [17], but the authors use information about the location of eye corners to initialize the template.

Eye corner detection has gained the attention of several research works, as it is relevant for multiple domains, such as biometrics, assisted driving systems, etc. In [19] the authors propose a new eye corner detection method in periocular images that simulate real-world data. First, the iris and the sclera region are segmented in the input image to determine the region of interest. Next, the eye corners are detected based on multiple features (response of Harris corners algorithm, the internal angle between the two corner candidates, their relative position in the ROI). The method gives accurate results on degraded data, proving its applicability in real-world conditions.

Recently sclera segmentation has shown to have a particular importance in the context of unconstrained, visible wavelength iris and sclera biometrics. Several works started to address this problem. In addition, sclera segmentation benchmarking competitions [20] were organized in order to evaluate the recent advances in this field and to attract researchers’ attention towards it.

The sclera region is usually segmented based on color information. In [21] the sclera region is roughly estimated by thresholding the saturation channel in the HSI color space; based on this segmentation, the region of interest for the iris is determined and the iris is segmented using a modified version of the circular Hough transform. Finally, the eyelid region that overlaps the iris is segmented using a linear Hough transform. 

Other methods [2,3,4] use more complex machine vision algorithms to segment out the sclera. In [4] Bayesian classifiers are used to decide whether the pixels belong to the sclera region or to the skin region, using the difference between the red and green, and blue and green channels from the RGB color space. In [2] three types of features are extracted from the training images: color features that illustrate the various relationships between pixels in different color spaces, Zernike moments and histogram of oriented gradients (HOG) features (the sclera has significant fewer edges than other regions around the eyes). A two stage classifier is trained to segment out the sclera region. The classifiers from the first stage operate on pixels, and the second stage classifier is a neural network that operates on the probabilities that are the output of the first stage. In [3] two classifiers are used: one for the sclera region and one for the iris region. The features used for the sclera classifier are Zernike moments and distinctive color features from different color spaces. The iris is localized based on three control points from the iris on the summation of two edge maps. Eyelids are segmented only near the iris area based on Canny edge detection and parabolic curve fitting.

## 3. Solution Description

### 3.1. Iris Segementation

**Figure 1:**
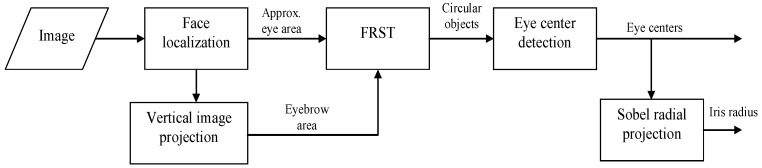
Iris segmentation algorithm.

The outline of the iris segmentation algorithm proposed in this paper is depicted in Figure 1. First, the face area is localized using the popular Viola-Jones face detector [22]. This method uses simple rectangular features (Haar-like features) and an efficient image representation, integral image transform, which allows computing the response of these features very quickly. The face region is segmented using the AdaBoost algorithm and a cascade of increasingly complex classifiers, which rapidly discards the background pixels.

**Figure 2:**
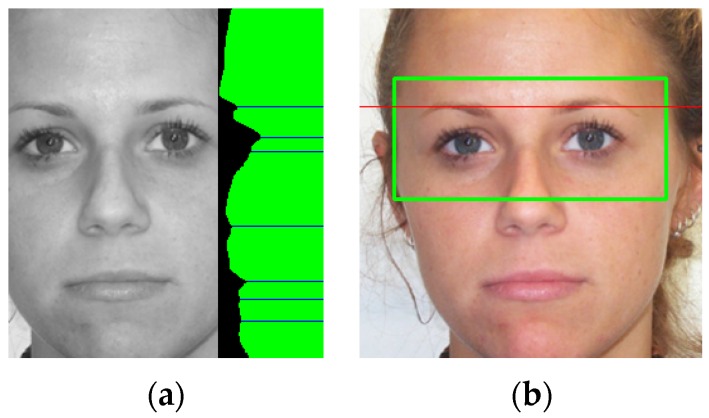
Eye region selection. (**a**) Cropped face image and vertical image projection (on the right). The local minima of the image projection are drawn with blue lines; and (**b**) the selected eye area (the green rectangle) and the estimated position of the eyebrow row (the red line).

On the face area, the eye region is computed using facial proportions. Additionally, the eyebrow area is roughly estimated using simple image projections. The width of the detected face image is cropped to 70% (15% of the image width are removed on each side) in order to assure that the background and the hair are removed from the image. The vertical image projection is computed on the gray scale intensity image and the first local minimum (projection valley) is selected as the rough estimation for the eyebrow region (Figure 2). 

#### 3.1.1. Iris Center Localization

Circular regions from the eye region are detected using a radial symmetry detector [23] (Fast Radial Symmetry Transform—FRST). The FRST is a gradient-based image operator that computes the role that each pixel *p* plays to the symmetry of the neighboring pixels at a radius *r*, by accumulating the orientation and magnitude contributions in the direction of the gradient.

**Figure 3:**
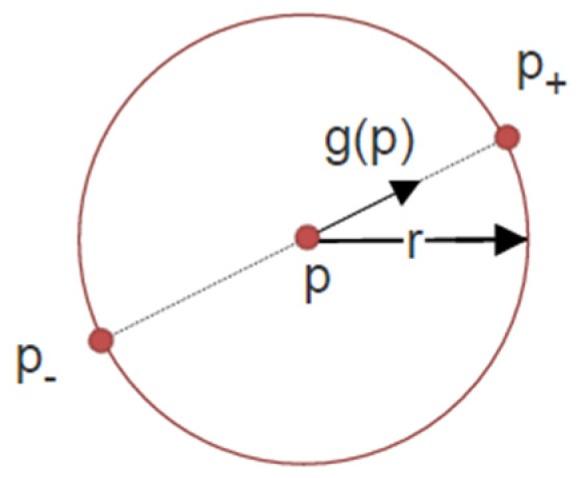
Positively- (*p_+_*) and negatively (*p*_−_)-affected pixels determined by the gradient element *g(p)* for a radius *r* (after [23]).

For each pixel *p*, a positively-affected pixel (*p_+_*) and a negatively-affected pixel *p*_−_ are computed (Figure 3); the positively-affected pixel is defined as the pixel the gradient is pointing to, at a distance *r* from *p*, and the negatively-affected pixel is the pixel the gradient is pointed away from at a distance *r* from *p*:

(1) $$p_{\pm }=p\;\pm round\left(\frac{g\left(p\right)}{\left|\left|g\left(p\right)\right|\right|}r\right)$$

where *g(p)* is the gradient vector at pixel *p*.

The orientation projection *O_r_* image and magnitude projection image *M_r_* are updated at each step based on the positively and negatively affected pixels:

(2) $$M_{r}\left(p_{\pm }\right)=\;M_{r}\left(p_{\pm }\right)\;\pm g\left(p\right),\;O_{r}\left(p_{\pm }\right)=\;O_{r}\left(p_{\pm }\right)\;\pm 1$$

Initially, the orientation projection and magnitude projection are set to zero.

For each radius *r* the symmetry transform is defined as the convolution (*):

(3) $$S_{r}=\;F_{r}\;\times A_{r}$$

where:

(4) $$F_{r}={\left|\left|\tilde{O_{r}}\;\left(p\right)\;\right|\right|}^{\left(\alpha \right)\;}\tilde{M_{r}}\left(p\right)$$

*A_r_* is a two dimensional Gaussian filter used to spread the symmetry contribution, $\tilde{O_{r}\;}$ and $\tilde{M_{r}}\;$ are the normalized orientation and magnitude projection images, and *α* is the radial strictness parameter. 

The full transform *S* is computed as the sum of symmetry contributions over all the radii considered:

(5) $$S=\;\underset{r}{\sum }Sr\;$$

The transform can be adapted to search only for dark or bright regions of symmetry: dark regions can be found by considering only the negatively affected pixels, while bright regions are found by considering only positively affected pixels when computing *O_r_* and *M_r_*.

As the iris region is darker than the surrounding area (the skin and sclera) only negatively-affected pixels are used to compute *O_r_* and *M_r_*. The search radii are determined based on anthropomorphic constraints: the eye width is approximately 1/5 [24] of the face width, and the ratio of iris width to eye width is about 0.42 in young adulthood and middle age and increases with age.

The centers of the irises are selected using geometrical and appearance constraints. 

The FRST image is scanned using a sliding window of 2 × *r_min_* size, where *r_min_* is the minimum radius for which the transform is computed, and the minimum intensity position from each window is retained. The minimum positions that are too close to each other (their distance is less than *r_min_*) are merged together, and the filtered minima are considered as possible iris candidates.

The iris centers are selected based on the circularity score from the symmetry transform image and geometrical conditions (the left iris center and right iris center must be positioned in the first and second half of the face width, respectively). Additionally, the confidence of the candidates that are positioned above the eyebrow area is penalized.

**Figure 4:**
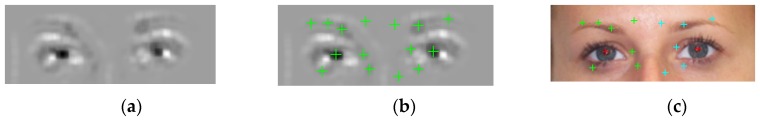
Iris center localization. (**a**) Symmetry transform on the eye region; (**b**) Possible iris center candidates in green; and (**c**) iris selection: left iris candidates in green, right iris candidates in cyan, and selected iris centers in red.

Figure 4 shows the result of the iris center localization module.

Finally, after the initial approximation of the iris center, a small neighborhood of (*r_min_*/2, *r_min_*/2) is analyzed and the center of the iris is constrained to the dark region of the pupil, similar to [12].

#### 3.1.2. Iris Radius Detection

The initial approximation of the iris radius range is based on geometrical face constraints. The problem now is to refine this rough estimation so that it best fits the real radius of the iris.

We use a vertical Sobel derivate on a blurred ROI around each eye in order to emphasize the strong transition between the iris and the sclera area. To eliminate the false transitions caused by potential image noise, the lowest *k*% of the gradients are ignored.

For each candidate radius *r* in the interval [*r_min_*, *r_max_*], a radial projection *proj(r)* is computed by adding the gradient values that are *r* pixels away from the iris center and span under the angles [*θ_min_*, *θ_max_*]:

(6) $$proj\left(r\right)=\overset{\theta_{max}}{\underset{\theta =\;\theta_{min}}{\sum }}sobel\left(c_{x}\pm \;r\cos\left(\theta \right),\;c_{y}\pm r\sin\left(\theta \right)\right)$$

where *sobel*(*x, y*) is the value of the gradient from the vertical Sobel image at (*x*, *y*) coordinates,$\left(c_{x},\;c_{y}\right)$ is the location of the iris center and $\left[\theta_{min},\;\theta_{max}\right]$ are the search angles.

The above *proj(r)* reaches its maximum values on the border between the sclera and the iris.

We limit the angular coordinate *θ* to an interval [*θ_min_*, *θ_max_*] because the border between the iris and the sclera yields the strongest transition, while the transition between the iris and the eyelids is less evident. Often the upper and lower parts of the iris are occluded by the eyelids. The value for [*θ_min_*, *θ_max_*] was set to [−45°, 45°] through trial and error experiments.

The radial projection is analyzed and the most prominent peak is selected as the iris radius:

(7) $$radius=\underset{r}{\text{argmax}}\;proj\left(r\right)$$

**Figure 5:**
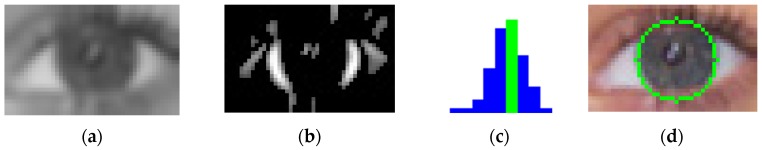
Iris radius computation. (**a**) Blurred eye region; (**b**) Vertical Sobel derivative (with *k* = 20% of the gradients ignored); (**c**) Radial projection of the image (the selected radius is depicted in green); and (**d**) the iris radius.

Figure 5 illustrates the steps of the iris radius computation algorithm.

### 3.2. Eye Shape Segmentation

**Figure 6:**
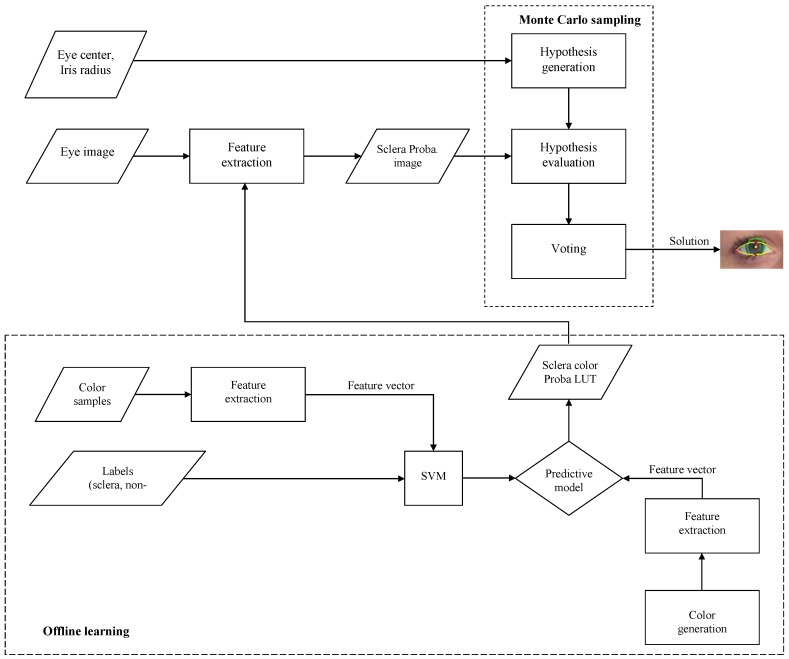
Eye shape detection algorithm.

The outline of the eye shape extraction procedure is depicted in Figure 6; the method is color-based and it exploits the high contrast between the sclera and its surrounding area.

An offline learning module is used to determine the probabilities of a color belonging to the sclera region. The output of this module is a look-up table (LUT) that stores the computed probabilities.

The eye shape extraction algorithm uses Monte Carlo sampling and a voting procedure to select the eyelid shape from a uniformly generated space of eye shape hypotheses. The *b* fittest individuals contribute to the selected eye shape.

#### 3.2.1. Sclera Probability LUT

A selected region around the eyes is transformed into a probability space using machine learning techniques to determine the probability of pixels belonging to the sclera region as described below.

The proposed approach adopts a pixel-based strategy for the classification and uses only color features: the hue component from the HSV color space, and the components of the RGB opponent color space. The RGB opponent color-space is a combination of three values based on the channels of the opponent color space [25]:

(8) $$\left(\begin{array}{c} O_{1}\\ O_{2}\\ O_{3} \end{array}\right)=\left(\begin{array}{c} \frac{R-G}{\sqrt{2}}\\ \frac{R+G-2B}{\sqrt{6}}\\ \frac{R+G+B}{\sqrt{3}} \end{array}\right)$$

The color information of the pixel is stored by *O*_1_ and *O*_2_ channels, while component *O*_3_ represents the intensity information.

The extracted chromatic features are used to train a linear Support Vector Machine (SVM) classifier which will be used to determine the probability of each pixel to belong to the sclera region.

**Figure 7:**
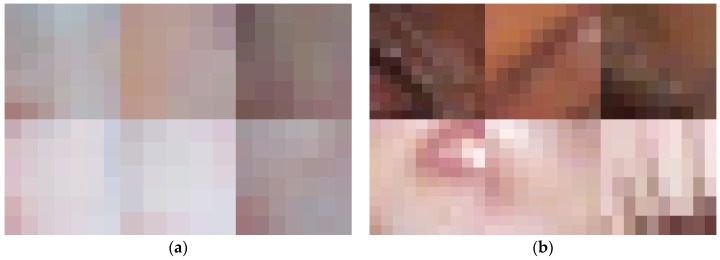
Sclera (**a**) and non-sclera (**b**) patches used to train the SVM classifier. The non-sclera patches are selected from the skin and eyelashes region.

The training of the SVM was performed on randomly-selected patches of approximately 10 × 10 pixels around the eye area from a publicly available face database [26]. Thirty patches were used for the non-sclera region and 25 patches for the sclera-region. Figure 7 shows some examples of sclera and non-sclera patches used for training.

Following the learning process Platt scaling [27] is performed to transform the binary output of the SVM classifier into probabilities. Other methods [3,4] use more complex features, such as edge information or Zernike moments, as features. Taking into account that the total number of colors that are representable in a 24 bits/pixel image is finite, a lookup table can be used to store the probability of each possible color. Therefore, our method has the advantage that it can pre-calculate the probability of each possible pixel, replacing the computation of the probability with a simple array indexing operation.

**Figure 8:**
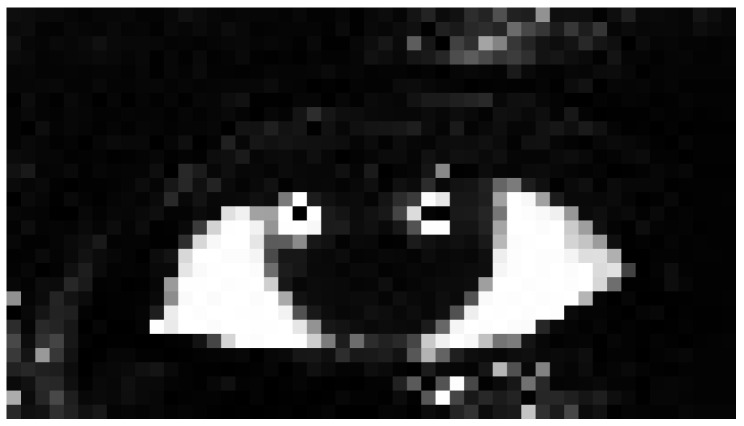
Sclera probability image (bright zones indicate a high sclera probability, while darker regions indicate a low sclera probability).

Figure 8 shows the output of the classifier of an input image.

#### 3.2.2. Eye Model

**Figure 9:**
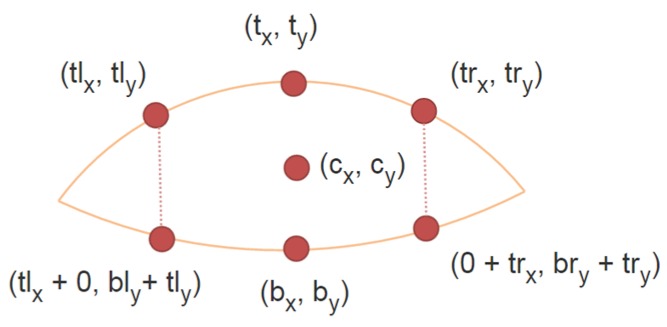
Eye model using seven control points (12 values) that generates two intersecting parabolas.

The exterior eye shape is represented using two parabolas, one for the upper eyelid and one for the bottom eye. The following shape definition vector, containing the coordinates of control points, is used to generate the shape of the eye:

(9) $$X=\;{\left[\begin{array}{cccccccccccc} c_{x} & c_{y} & t_{x} & t_{y} & b_{x} & b_{y} & tl_{x} & tl_{y} & tr_{x} & tr_{y} & bl_{y} & br_{y} \end{array}\right]}^{T}$$

where (*c_x_*, *c_y_*) are the (*x*, *y*) coordinates of iris center, (*t_x_*, *t_y_*) is the middle point on the top eyelid, (*b_x_*, *b_y_*) is the middle point on the bottom eyelid. For the top eyelid we define two other control points (*lt_x_*, *lt_y_*) and (*rl_x_*, *rl_y_*) to the left and right of the middle point. These landmarks have two corresponding on the bottom eyelid that have the same *x* coordinates as the first two points, their y coordinates are defined by the offset values *bl_y_* and *br_y_* (Figure 9). 

The coordinates of the control points of the eye external shape are expressed relatively to the eye center in order to make the model invariable to translation.

#### 3.2.3. Shape Matching

Using the described model, multiple hypotheses are generated and the sclera probability image is used to determine how well a hypothesis conforms to the input image. Ideally, the eyelid parabola should be positioned exactly on the boundary between sclera pixels (brighter region) and non-sclera pixels (darker region).

**Figure 10:**
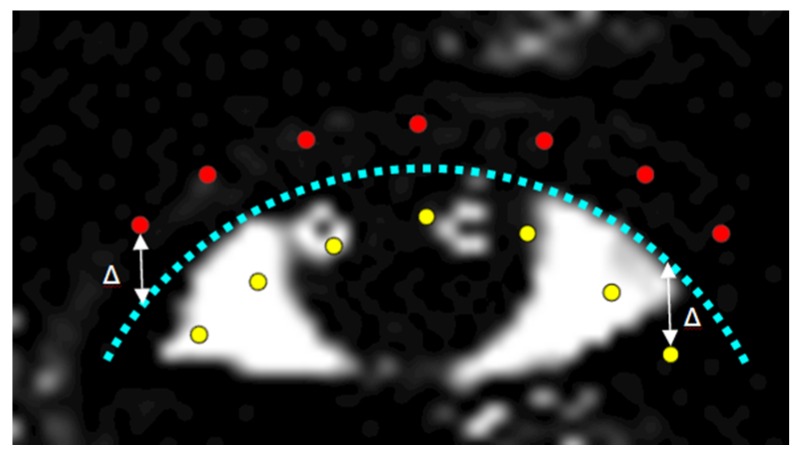
Eyelid shape matching (for the top eyelid only). The dotted line represents the current hypothesis; the positive pixels are depicted in yellow and the negative pixels in red.

In order to express the above assumption we use a similar approach as in [28]. Two sets of pixels at Δ distance away from the current hypothesis are considered: the positive pixels (*p_+_*) which are the pixels that belong to the sclera region and negative pixels (*p*_−_) which are the pixels from the skin and eyelashes zone (Figure 10). 

The score of a hypothesis is defined as:

(10) $$\omega =\;\frac{\alpha \;\sum_{\Delta }p_{+}-\;\beta \;\sum_{\Delta }p_{-}}{\alpha +\;\beta }$$

where *α* and *β* (*α* + *β* = 1) are the weights that control the influence of positive and negative pixels, respectively. 

We assign the same contribution to both sets of pixels, and therefore both weights, *α* and *β*, are set to 0.5. The proposed metric computes the average difference between the probability values of the inner part of the eyelid curve, which must fit inside of the sclera region and, thus, expected to have a high sclera probability value, and the values of the outer area of the curve, which must fit on the outside of the eye and, thus, expected to have a low sclera probability value. 

Several variations of this metric were tested: to consider only the pixels from the set {0, Δ}, that is the pixels at Δ distance away from the current hypothesis, or to examine all the pixels in the in interval [1, Δ]. The best results were obtained by computing the metric over all the pixels in the interval [1, Δ]. 

The value of Δ is set to a value that is proportional to the iris radius (in our case 5 pixels). A lower value of this parameter does not propagate enough the transition between the sclera and the non-sclera regions and is very sensitive to noise, while a higher value could lead to missing the region of interest.

#### 3.2.4. Eye Shape Selection

To find the exterior shape of the eye, a simple Monte Carlo sampling is used. Monte Carlo algorithms form a wide class of methods that are based on repeated resampling of random variables over an input domain, which defines the phenomena in question. These methods have been successfully used to solve optimization problems with complex objective functions.

*N* hypotheses are uniformly generated over the input domain defined by the iris center position and the iris radius. Each hypothesis is matched over the input image using the method described in Section 3.2.3. 

The solution is selected by considering the best *b*% hypotheses: each one of these shapes contributes to the solution by a weight that is directly proportional to its fitness value. In other words, the position for each control point of the result is computed as:

(11) $$\left(x,\;y\right)=\;\frac{\sum \omega_{i}\cdot p_{i\;}\left(x,y\right)}{\sum \omega_{i}}$$

where *s*(*x*, *y*) represents the (*x*, *y*) coordinates of a control point from the solution, $p_{i}\left(x,y\right)$ and $\omega_{i}$ are the position and the score function of the *i*th hypothesis that votes for the solution.

**Figure 11:**
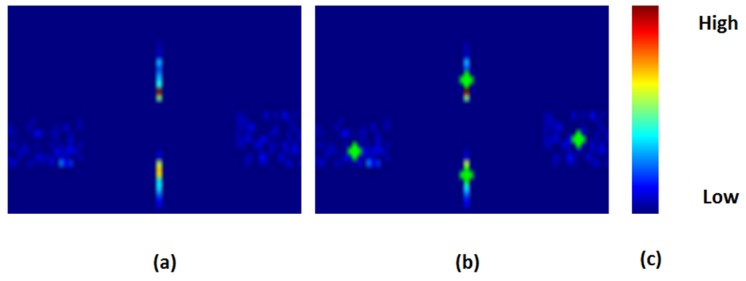
Eye shape voting procedure. (**a**) The distribution of the markers obtained through the voting of the fittest *b* = 30% hypotheses; cold colors represent smaller values and warm color represent higher values; (**b**) The solution control points (marked in green) computed as the weighted average of the fittest hypotheses. The intersection between the lower and the upper parabola is computed so that the solution can be expressed through the eye corners and two points on the upper and lower eyelids; and (**c**) color scale.

Figure 11 graphically illustrates the voting process of the proposed algorithm using color maps. 

The optimal value for *N* was heuristically determined to 200 samples. From our experiments we determined that increasing the number of samples of the Monte Carlo sampling does not cause a relevant increase in the accuracy of the algorithm. A value for *N* lower than 100 has a negative influence on the accuracy of the algorithm. The reported results in Section 4 were obtained by generating *N* = 200 hypotheses and considering the fittest 30% hypotheses in the voting process.

**Figure 12:**
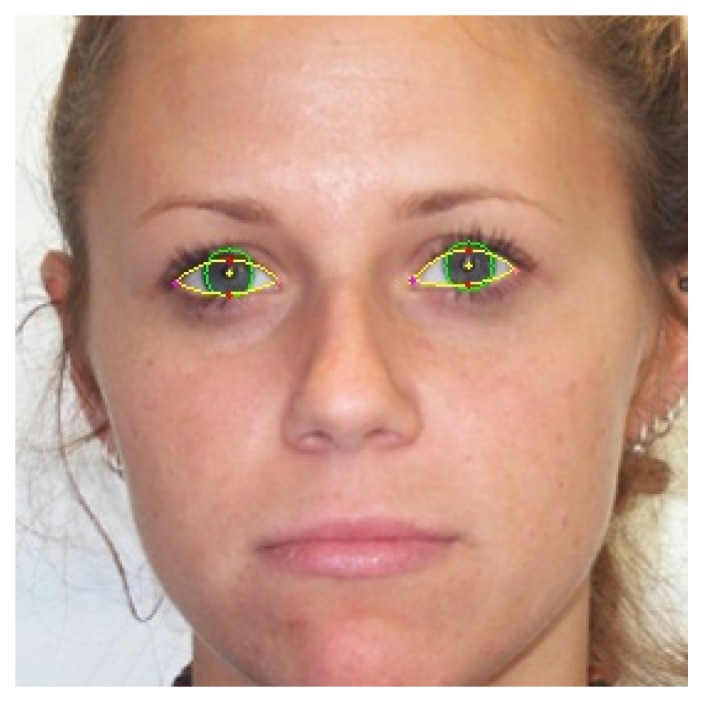
Eye shape segmentation results.

The result of the eye shape segmentation algorithm is depicted in Figure 12.

## 4. Results and Discussion

### 4.1. Iris Center Localization

The metric used to validate the performance of the eye center localization is the relative error introduced in [9]: the error obtained by the worst of both eye estimators, normalized with the distance between the eye centers:

(12) $$wec\leq \;\frac{\max\left(\|C_{l}-\hat{C_{l}}\;\|,\;\|C_{r}-\hat{C_{r}}\;\|\right)}{\left\|C_{l}-\;C_{r}\right\|}$$

where $C_{l}$, $C_{r}$ are the positions of the left and right iris centers, and $\hat{C_{l}}$, $\hat{C_{r}}$ are the positions of the estimated left eye and right iris centers.

This metric is independent of the image size. Based on the fact that the distance between the inner eye corners is approximately equal to the width of an eye, the relative error metric has the following properties: if *wec* ≤ 0.25 the error is less than or equal to distance between the eye center and the eye corners, if *wec* ≤ 0.10 the localization error is less than or equal to the diameter of the iris, and finally, if *wec* ≤ 0.05 the error is less than or equal to the diameter of the pupil. 

In addition two other metrics were implemented like suggested in [12]: *bec* and *aec* which define the lower and the averaged error, respectively:

(13) $$bec\;\leq \;\frac{\min\left(\|C_{l}-\hat{C_{l}}\;\|,\|\;C_{r}-\hat{C_{r}}\;\|\right)}{\left\|C_{l}-\;C_{r}\right\|}$$

(14) $$aec\leq \;\frac{\text{avg}\left(\left\|C_{l}-\hat{C_{l}}\;\|,\|C_{r}-\hat{C_{r}}\;\right\|\right)}{\left\|C_{l}-\;C_{r}\right\|}$$

where min() and avg() are the minimum and the average operators. 

The proposed iris center localization algorithm does not use any color information, only the sclera segmentation part. For comparison purposes, our iris center localization method is evaluated on the BIO-ID face database [9], one of the most challenging eye databases, which has been used for the validation of numerous eye localization methods. The dataset reflects realistic image capturing conditions, featuring a large range of illumination conditions, background and face size and many state of the art methods were tested on this dataset. The database contains 1521 grey-scale images of 23 persons, captured during different sessions in variable illumination conditions. Moreover, some of the subjects in the database wear glasses, in some images the eyes are (half) closed or the eyes are occluded by strong specular reflections on the glasses. The resolution of the images is low 384 × 286 pixels. This dataset has been widely used to evaluate eye center localization methods and, therefore, it allows us to benchmark the results of our algorithm with prior work.

**Figure 13:**
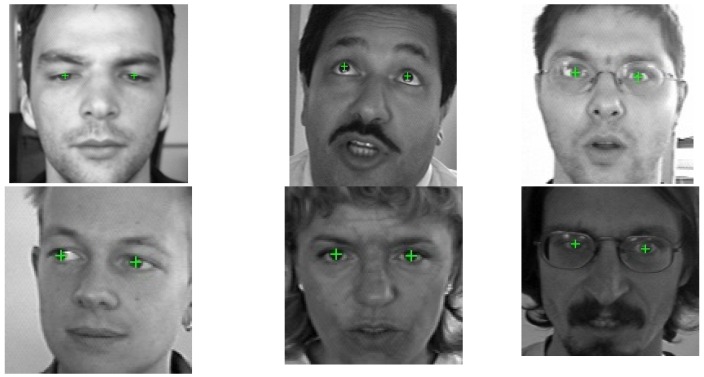

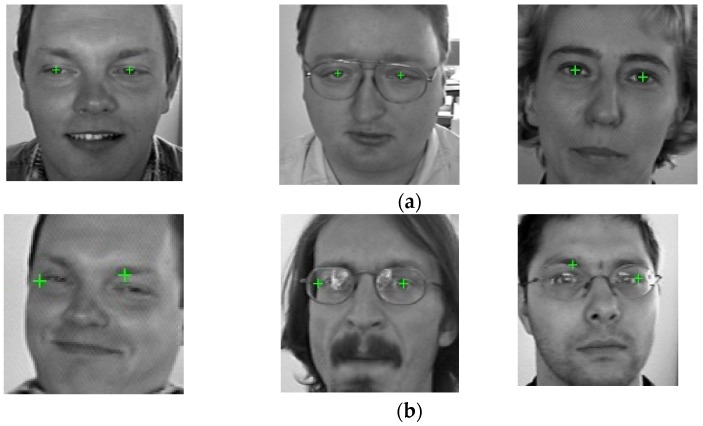
Iris center localization results on the BIO-ID face database. (**a**) Iris center localization results; and (**b**) iris center localization results—failure cases.

**Figure 14:**
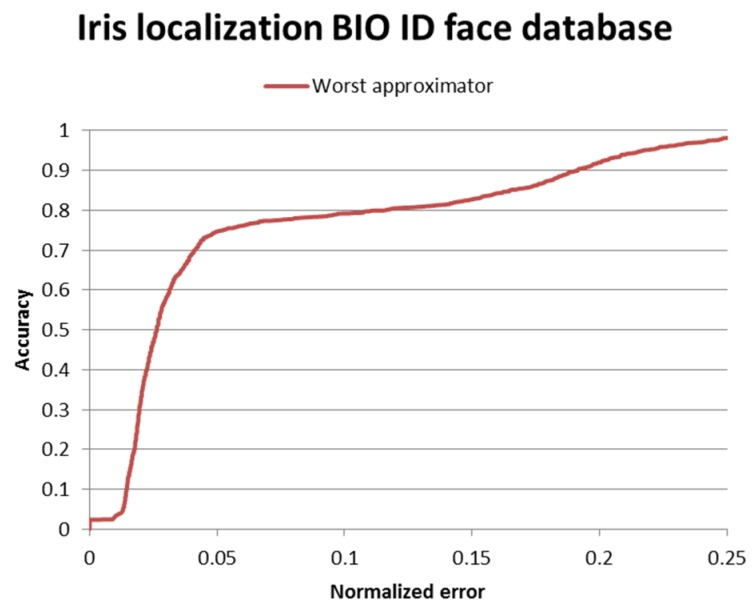
Iris center localization results on the BIO-ID face database.

Results on the BIO-ID face database are depicted in Figure 13 and the ROC curve is depicted in Figure 14.

The comparison of our method with other state of the art papers is shown in Table 1. If the performance for the normalized error ∈ {0.05, 0.10, 0.25} was not mentioned explicitly by the authors, we extracted the values from the performance curves; these values are marked with * in the table.

In the case of pupil localization (*wec* ≤ 0.05) the proposed method is outperformed only by [12]. In the case of eye localization (*wec* ≤ 0.25) our method outperforms the other works. However, in the case of *wec* ≤ 0.10 the proposed algorithm is outperformed by three other state of the art methods [8,12,15]. This is due to the fact that our eye center localization algorithm relies mostly on circularity constraints and the BIO-ID face database contains multiple images where the eyes are almost closed, in which case the circularity of the iris cannot be observed. Therefore, the accuracy of the algorithm is impaired. The transition between the cases *wec* ≤ 0.05 and *wec* ≤ 0.25 is smoother because in multiple images of the database the circularity of the iris is not observable due to occlusions and closed eyes. To sum up, the proposed algorithm yields accurate results (*wec* ≤ 0.05 in 74.65% of the images) for the images where the iris is visible, and acceptable results otherwise.

However, our method was designed for use cases, such as biometry, optometry, or human emotion understanding, in which the face is the main component under analysis and the facial region has medium to good quality. The BIO-ID face database is not adequate for this purpose due to the low quality of the images. We tested our algorithm on this database so that we can compare with other methods.

**Figure 15:**
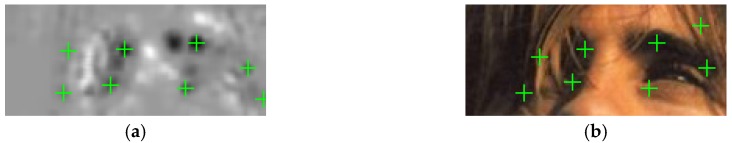
Failure cases of eye center localization. (**a**) Symmetry transform image and selected eye candidates; and (**b**) selected eye candidates (on the input image).

The proposed iris localization method based on the accumulation of first order derivatives obtains accurate results if the iris is relatively visible in the input image. The search region for the eyes often contains other elements, such as eyeglasses, eyebrows, hair, etc., and, if the iris is occluded in some way (semi-closed eyes or strong specular reflections of the eyeglasses), these external elements could generate a higher circularity response than the actual iris (Figure 15). 

We try to filter out these false candidates by imposing appearance constraints—the pupil center must be darker than the surrounding area, so the result of the symmetry transform image is weighted by the inversed, blurred gray-scale image, and several geometrical constraints: separation of the left and right eye candidates, penalization of the eye candidates that are too close to the eyebrow area. Considering that we are interested in obtaining a pair of eye centers, we have also included a metric that models the confidence of the candidates as a pair. The score of a pair is weighted by a Gaussian function of the inter-pupillary distance normalized by the face width having as mean the average ratio between the inter pupillary distance and the face width [24]. However, this method was designed mainly for application domains (such as optometry, human-computer interaction, etc.) in which the user is cooperative and the eye is visible in the input image and therefore these exaggerated occlusions are less likely to occur.

As we will further demonstrate, the performance of the algorithm increases with the quality of the image, while keeping the computational power low (on average, the eye center localization algorithm takes six milliseconds on an Intel Core i7 processor). 

To test the proposed method we annotated a publicly-available face database [26], created by the University of Michigan Psychology department. In the rest of the paper we will refer to this database as University of Michigan Face Database (UMFD). The database comprises facial images of 575 individuals with ages ranging from ages 18 to 93 and is intended to capture the representative features of age groups across the lifespan. The dataset contains pictures of 218 adults age 18–29, 76 adults age 30–49, 123 adults age 50–69, and 158 adults age 70 and older. 

**Figure 16:**
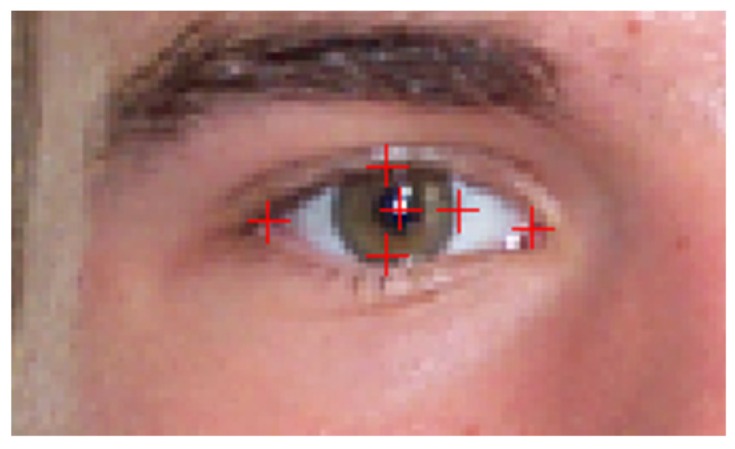
Eye landmarks used on the annotation of University of Michigan Face Database.

Six points were marked on each eye: the center of the pupil, the eye corners, the top and the bottom eyelids and a point on the boundary of the iris (Figure 16). The database annotation data can be accessed from [5]. The structure of the annotation data is detailed in Appendix A.

**Figure 17:**
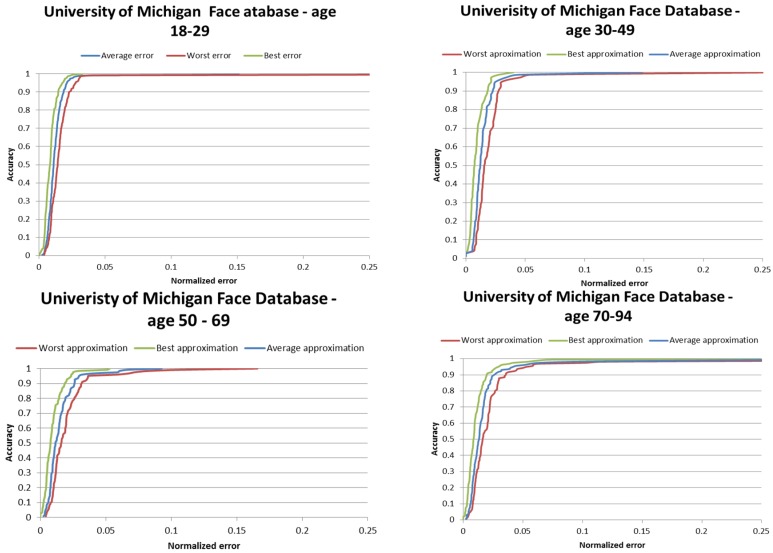
Performance of the iris center localization algorithm on the University of Michigan Face Database.

The ROC curves for the iris center localization on the age groups from the University of Michigan database are illustrated in Figure 17 and Table 2 shows the eye center localization results on this database.

From the Table 2 it can be noticed that the on medium quality facial images (640 × 480) the performance of the proposed algorithm is highly increased: in 96.30% of the cases, the worst of the two eye center approximations falls into the pupil area.

The accuracy of the algorithm is lower (93.63%) for older subjects of ages between 70 and 94 years old due to the fact that a lower portion of the iris is visible and its circularity cannot be observed.

In conclusion, the proposed eye localization method proves to be efficient on all the use cases considered: pupil localization (*wec* ≤ 0.05), iris localization (*wec* ≤ 0.10), and eye localization (*wec* ≤ 0.25).

### 4.2. Iris Radius Computation

To evaluate the accuracy of the iris radius estimation algorithm we compute the following normalized errors:

(15) $$aer=\;\frac{\left|\hat{rr}-rr\right|+\left|\hat{rl}-rl\right|}{rl+rr}$$

(16) $$wer=\;\frac{2\cdot max\left(\left|\hat{rr}-rr\right|,\left|\hat{rl}-rl\right|\right)}{rl+rr}$$

(17) $$ber=\;\frac{2\cdot min\left(\left|\hat{rr}-rr\right|,\left|\hat{rl}-rl\right|\right)}{rl+rr}$$

where *rl* and *rr* are the radiuses of the left and right iris, and $\hat{rr}$ and $\hat{rl}$ are the estimated radiuses of the right and left eye, respectively. The *wer* (worst error radius) metric represents the radius error for the worst radius estimator, the *aer* (average error radius) is the average radius error for the left and right iris and *ber* (best error radius) is the radius error for the best radius estimator. The functions are normalized by the average of the correct irises radius.

Table 3 shows the performance of the iris radius computation algorithm on the different age groups from the University of Michigan Face Database.

On average, the normalized *aer* value is 0.0991; in other words, the average error of the iris radius is less that 10% of the actual radius. Taking into account the fact that the iris radius has approximately 12 pixels on the images from the database, the magnitude of the error is about 1–2 pixel.

### 4.3. Eye Shape Segmentation

To evaluate the eye shape segmentation algorithm, we used several statistical measures of the performance by analyzing the proportion of pixels that are assigned to the eye or non-eye region.

We computed the number of true positives (*TP*), true negatives (*TN*), false positives (*FP*), and false negatives (*FN*), by comparing the results of the algorithm with the ground truth from the test databases. The terms true (*T*) and false (*F*) refer to ground truth and the terms positive (*P*) and negative (*N*) refer to the algorithm decision. Based on these values, the following statistical measures were determined:

(18) $$sensitivity=\;\frac{TP}{TP+FN}$$

(19) $$specificity=\;\frac{TN}{TN+FP}$$

(20) $$accuracy\;=\;\frac{TP+TN}{TP+\;TN+FP+FN}$$

Sensitivity (or recall) is a measure of the proportion of eye pixels that are correctly identified and indicates the algorithm’s ability to correctly detect the eye region; specificity (or true negative rate) measures the proportion of non-eye pixels that are correctly identified as such and relates to the algorithm’s ability to rule out the pixels that do not belong to the eye region. In other words sensitivity quantifies the algorithm ability to avoid false negatives, while specificity quantifies its ability to avoid false positives.

**Figure 18:**
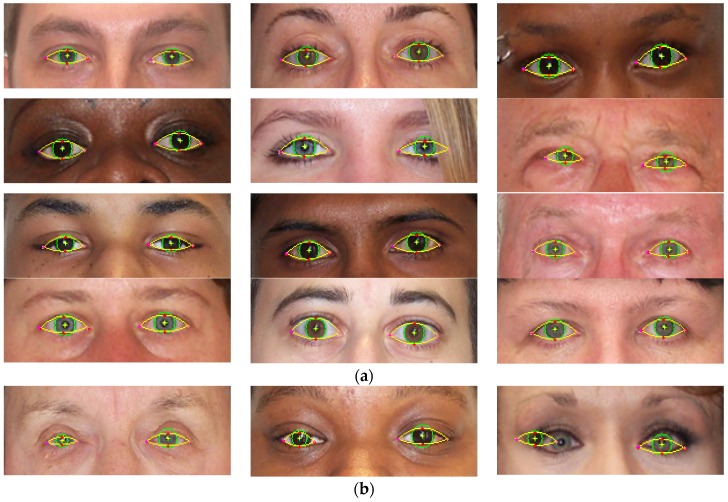
Eye shape segmentation results on University of Michigan Face Database. (**a**) Eye shape extraction results; and (**b**) failure cases.

Eye shape segmentation results are illustrated in Figure 18. The detected iris is marked with a green circle and the upper and lower eyelid parabolas are depicted in yellow.

**Figure 19:**
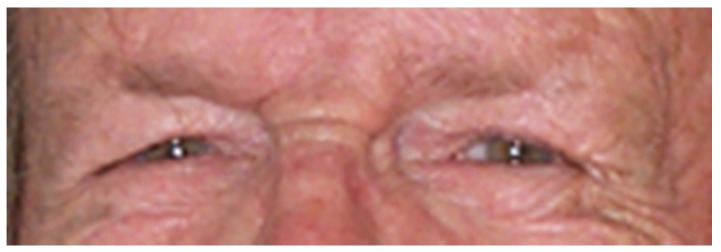
The effect of aging over the eye region.

From the results on University of Michigan Face Database it can be noticed that the accuracy of the algorithm is decreasing with age. On older subjects the sclera portion of the eye becomes less visible due to eyelid ptosis and skin excess around the eyes. The shape of eyelid is distorted and it can no longer be estimated with a parabola (Figure 19). In addition, sclera color quality degradation [29] is a well-known effect of aging on the eyes that influences the performance of the algorithm. The loss of performance is about 1%–2% in average.

The results of the eye shape segmentation algorithm are strongly dependent on the accuracy of the eye center localization. For example, in the last image from Figure 18 it can be seen that the performance of the algorithm is impaired due to the wrong eye center estimation.

To the best of our knowledge, full eye segmentation methods that we can compare with have not been reported previously in the specialized literature. An older work [17] uses a deformable template to find the full shape of the eye, but only the run time of the algorithm is reported.

**Figure 20:**
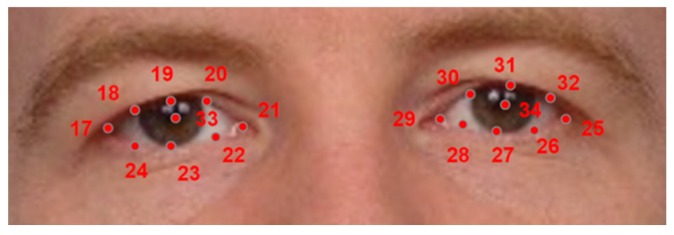
The landmarks from the IMM face database denoting the eye region.

The algorithm was also tested on an image database that is independent from our training set, the IMM frontal face database [30], which contains 120 facial images of 12 different subjects. All of the images are annotated with 73 landmarks that define the facial features for the eyebrows, nose, and jaws. The contour of each eye is marked with eight points (Figure 20). 

**Figure 21:**
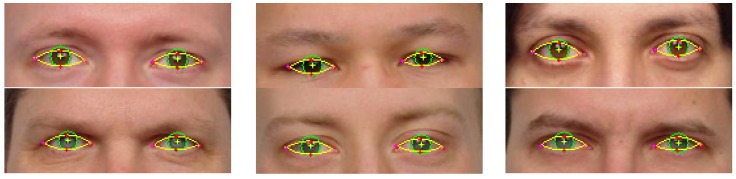
Eye shape segmentation on the IMM face database.

Results on the IMM face database are depicted in Figure 21 and the numerical scores are shown in Table 4.

Labeled Face Parts in the Wild (LFPW) [10] is a large, real-world dataset of hand labeled images, acquired from Internet search sites using simple text queries. The images from the dataset are captured in unconstrained environments and contain several elements that can impair the performance of the algorithm: the eyes are occluded by heavy shadowing or (sun-) glasses, hats, hair, etc., some faces contain a lot of make-up and present various (theatrical) facial expressions. The only precondition of the images is that they are detectable by a face detector. Each image was annotated with 29 fiducial points. The images contain the annotation of three different workers and the average of these annotations was used as the ground truth. Due to copyright issues, the image files are not distributed, but rather a list of URLs is provided from which the images can be downloaded. Therefore, not all the original images are still available, as some of the image links have disappeared. We have downloaded all the images that were still accessible (576 images) from the original set of images and we evaluated the performance of our method on this dataset. 

The mean errors of our algorithm compared to other state of the art works and a commercial off the shelf (COTS) system [10] are shown in Table 5.

From Table 5 it can be noticed that our method is comparable with the COTS system and [11], but [10] is more accurate. However, [10] detects 29 fiducial points on the face and the total processing time for an image is 29 s. Our method takes on average 20 ms to find all of the six landmarks around the eyes, being several orders of magnitude faster than [10].

For the eye center localization, the average normalized error is in average 0.0426. A normalized error less than 0.05 implies that the detected iris center is within the pupil area. Therefore, our method has a good performance even on images that are degraded due to capturing conditions.

**Figure 22:**
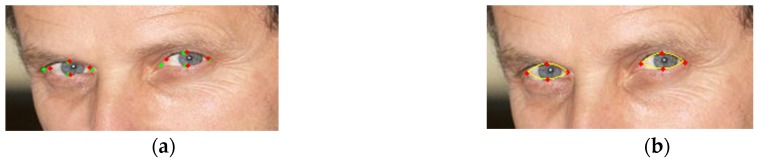
Difference between the annotated eye corners and the detected sclera landmarks. It can be noticed that even if the sclera is correctly segmented, there is a larger distance between the annotated eye corners and the sclera landmarks. (**a**) Annotated landmarks on LFPW database (in green) and detected sclera landmarks (in red); and (**b**) segmented sclera region.

For the sclera landmarks, the algorithm yields larger localization errors: on average the normalized distance between the sclera landmarks and the annotated landmarks is 0.0607. First, we note a difference of semantics between the annotated landmarks and the result of our algorithm: the proposed method is intended to segment the sclera region as accurately as possible and not to detect the position of the eye corners. While in some cases the sclera landmarks can determine the exact eye corners this cannot be generalized. For example, from Figure 22 it can be noticed that the sclera region is correctly segmented but the distance between the annotated left eye inner corner and the corresponding sclera landmarks is large; the normalized error between these two points is 0.0708. 

In addition, some of the images in the database contain sunglasses that totally obstruct the eyes and in some other images the sclera is not visible, due to the low image resolution, and cannot be accurately segmented even by a human operator. The problems targeted by our solution are iris and sclera segmentation; in order to solve these problems the features under consideration must be visible in the input image. 

**Figure 23:**
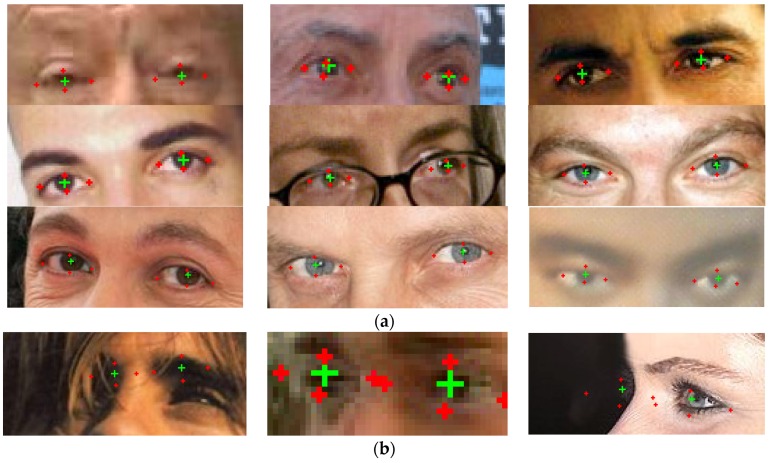
Results on the LFPW database. The eye centers are depicted with a green cross and the landmarks used to generate the shape of the eyelids are marked red crosses. (**a**) Results; and (**b**) failure cases.

Figure 23 shows the results of our method on some images from the LFPW database. Due to the smaller resolution of the images, we only draw the landmarks (eye centers and the landmarks used to generate the eyelid parabolas).

The main application domains targeted by our method are optometry and ophthalmology, augmented reality, and human-computer interaction, where the quality of the images is usually medium to good and the user is cooperative. 

**Figure 24:**
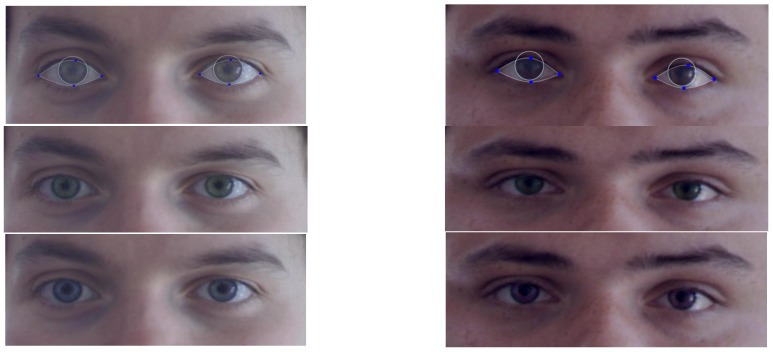
Demonstrator application—virtual contact lens simulator. On the first row: detection: detection result (iris circle and sclera segmentation). On the next two rows: different contact lens simulations: natural green, dark green, ocean blue, and violet.

The method is integrated into a virtual contact lens simulator application and into a digital optometric application that measures the iris diameter and the segment height (the vertical distance in mm from the bottom of the lens to the beginning of the progressive addition on a progressive lens) based on facial images. Snapshots of the virtual contact lens simulator application are presented in Figure 24: different contact lenses colors are simulated: dark-green, hazel, natural green, and ocean blue respectively.

The average execution time for the full eye segmentation algorithm (the iris and the eyelids) is on average 20 ms on a on an Intel Core i7 processor on 640 × 480 resolution images.

## 5. Conclusions 

This paper presents a fast eye segmentation method that extracts multiple features of the eye region: including the center of the pupil, the iris radius, and the external shape of the eyes. Our work has superior accuracy compared to the majority of the state of the art methods that measure only a subset of these features. 

The eye features are extracted using a multistage algorithm: first the iris center is accurately detected using circularity constraints and, on the second stage, the external eye shape is extracted based on color and shape information through a Monte Carlo sampling framework.

Compared to other state of the art works our method extracts the full shape of the eye (iris and full eyelid boundaries), and we consider that it is sufficiently generic so that it has applications in a variety of domains: optometry, biometry, eye tracking, and so on.

Extensive experiments were performed to demonstrate the effectiveness of the algorithm. Our experiments show that the accuracy of the method is dependent to the image resolution: increasing the image quality leads to an increase of accuracy without excessively increasing the computation time.

Future work will include increasing the accuracy of the estimated eye shape using more measurement cues (like corner detectors for the eye corners) and tracking of the iris centers. By tracking the iris centers the detection system will benefit by reducing the detection failure in the case of illumination changes or when one of the irises is not fully visible. Additionally, we intend to use other curves for representing the eyelid shapes, such as third degree polynomials, spline, or Bezier curves, in order to increase the performance of the eye shape segmentation. 

## Figures and Tables

**Table 1:** Iris center localization accuracies compared to the state-of-the-art methods.

Method	(wec≤0.05)	(wec≤0.10)	(wec≤0.25)
Timm et al. [12]	82.5%	93.4%	98.0%
Hassaballah et al. [15]	37% *	84% *	94.3%
Cristianace et al. [8]	57% *	96%	97.1%
Jerosky et al. [9]	38.0% *	78.8% *	91.8%
Proposed method	74.6594%	79.1553%	98.09%

* The performance was not explicitly stated by the authors and it was read from the ROC curve.

**Table 2:** Iris center localization results on the University of Michigan face database.

Dataset	Iris Center Localization Accuracy								
	Error ≤ 0.05			Error ≤ 0.10			Error ≤ 0.25		
	*wec (%)*	*aec (%)*	*bec (%)*	*wec (%)*	*aec (%)*	*bec (%)*	*wec (%)*	*aec (%)*	*bec (%)*
UMFD age 18–29	99.08	99.08	100	99.08	98.08	100	99.08	100	100
UMFD age 30–49	97.36	98.68	100	98.68	98.68	100	98.68	100	100
UMFD age 50–69	95.12	96.74	98.37	98.37	100	100	100	100	100
UMFD age 70–94	93.63	95.54	97.45	96.81.	98.09	99.36	98.09	98.72	99.36
Average	96.30	97.51	98.96	98.71	98.71	99.84	98.96	99.68	99.84

**Table 3:** Iris radius computation results on the University of Michigan Face Database.

Dataset	*aer*	*wer*	*ber*
University of Michigan Face Database (age18–29)	0.0903	0.1269	0.053
University of Michigan Face Database (age 30–49)	0.0885	0.1276	0.0495
University of Michigan Face Database (age 50–69)	0.1247	0.1721	0.0774
University of Michigan Face Database (age 70–94)	0.0928	0.1361	0.0495
Average	0.0991	0.1406	0.05735

**Table 4:** Performance of the eye shape segmentation algorithm the UMFD and IMM datasets.

Dataset	Eye Shape Segmentation Results		
	Sensitivity	Specificity	Accuracy
UMFD (age 18–29)	87.61%	90.02%	88.77%
UMFD (age 30–49)	90.75%	83.29%	86.57%
UMFD (age 50–69)	84.35%	83.06%	83.58%
UMFD (age 70–94)	84.76%	82.41%	83.41%
IMM Face Database	86.3%	90.13%	87.93%

**Table 5:** Mean error normalized by the inter-pupillary distance.

Landmark	[10]	[11]	COTS [10]	Our Method
Left eye error	**0.024**	n/a	n/a	0.0412
Right eye error	**0.027**	n/a	n/a	0.0440
Left eye inner corner	**0.0285**	0.072	**0.048**	0.0677
Left eye outer corner	**0.035**	0.077	0.076	0.0701
Left eye upper eyelid	**0.027**	n/a	n/a	0.0503
Left eye bottom eyelid	**0.03**	n/a	n/a	0.0564
Right eye inner corner	**0.028**	**0.055**	**0.047**	0.0672
Right eye outer corner	**0.033**	0.084	0.07	0.0694
Right eye upper eyelid	**0.027**	n/a	n/a	0.0486
Right eye bottom eyelid	**0.028**	n/a	n/a	0.0562

**Figure A1:**
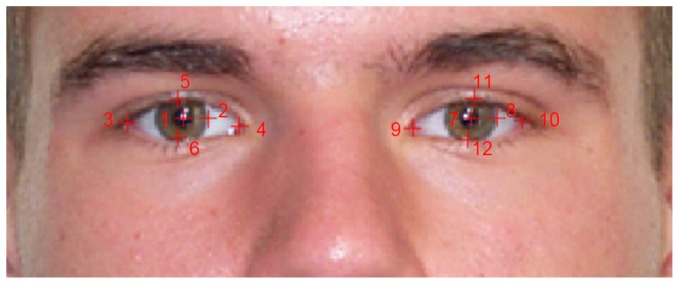
The 12 annotation points used to define the eye features.

## Supplementary Materials

The eye feature annotation of the UMFD dataset is available online at https://drive.google.com/folderview?id=0Bwn_NrD78q_Td3VjOXA2c183UHM&usp=sharing.

## Abbreviations

The following abbreviations are used in this manuscript:

ROI	Region of interest
CLM	Constrained Local Models
HOG	Histogram of Oriented Gradients
FRST	Fast Radial Symmetry Transform
LUT	Look Up Table
SVM	Support Vector Machine
UMFD	University of Michigan Face Database
TP	True Positives
TN	True Negatives
FP	False Positives
FN	False Negatives
LFPW	Labeled Face Parts in the Wild database
COTS	commercial off the shelf

## Appendix A

This section describes the annotation format of the University of Michigan Face Database [26]. This dataset contains a large number of faces of individuals throughout the adult lifespan (from 18 to 94 years) and it includes representative faces from both younger and older subjects.

A detailed description regarding the data acquisition procedure and the breakdown of participants by age, race, and gender can be found in [26]. Access to the database is restricted to academic researchers.

This work provides only the database annotation files; to request access to the images please contact the authors of [26].

For each image from the dataset, the annotation data is stored into a .*pts* file with the same base-name the image; for example, the annotation file for the image *EMWmale19neutral*.*bmp* is stored in the file *EMWmale19neutral.bmp.pts*.

All photos were annotated with 12 landmarks around the eyes. Table A1 specifies the correspondence between annotation landmarks and eye features and Figure A1 illustrates the exact localization of each one of these points.

**Table A1:** The 12 landmarks defining the eye features.

Eye Type	Eye Feature	Landmark #
Left eye	Iris center	1
	Iris radius	2
	Eye external corner	3
	Eye interior corner	4
	Top eyelid	5
	Bottom eyelid	6
Right eye	Iris center	7
	Iris radius	8
	Eye external corner	9
	Eye interior corner	10
	Top eyelid	11
	Bottom eyelid	12

A *.pts* file is a simple text file structured as a set of line separated by a new line character. The layout of the file is as follows:

- The first line contains the total number *n* of points annotated in the image (in this case *n = 12*);
- The following lines, ranging from *k = 2* to *n + 1* contain the coordinates of the (*k − 1)^th^* landmark (one point per line)

The annotation can be freely used for education and research purposes.
